# Normothermic machine perfusion of ischaemically damaged porcine kidneys with autologous, allogeneic porcine and human red blood cells

**DOI:** 10.1371/journal.pone.0229566

**Published:** 2020-03-10

**Authors:** Merel B. F. Pool, Loes Hartveld, Henri G. D. Leuvenink, Cyril Moers

**Affiliations:** Department of Surgery–Organ Donation and Transplantation, University Medical Center Groningen, Groningen, the Netherlands; University Medical Center Utrecht, NETHERLANDS

## Abstract

In porcine kidney auto-transplant models, red blood cells (RBCs) are required for ex-vivo normothermic machine perfusion (NMP). As large quantities of RBCs are needed for NMP, utilising autologous RBCs would imply lethal exsanguination of the pig that is donor and recipient-to-be in the same experiment. The purpose of this study was to determine if an isolated porcine kidney can also be perfused with allogeneic porcine or human RBCs instead. Porcine kidneys, autologous and allogeneic blood were obtained from a local slaughterhouse. Human RBCs (O-pos), were provided by our transfusion laboratory. Warm ischaemia time was standardised at 20 minutes and subsequent hypothermic machine perfusion lasted 1.5–2.5 hours. Next, kidneys underwent NMP at 37°C during 7 hours with Williams' Medium E and washed, leukocyte depleted RBCs of either autologous, allogeneic, or human origin (n = 5 per group). During perfusion all kidneys were functional and produced urine. No macroscopic adverse reactions were observed. Creatinine clearance during NMP was significantly higher in the human RBC group in comparison with the allogeneic group (P = 0.049) but not compared to the autologous group. The concentration of albumin in the urine was significantly higher in the human RBC group (P <0.001) compared to the autologous and allogeneic RBC group. Injury marker aspartate aminotransferase was significantly higher in the human RBC group in comparison with the allogeneic group (P = 0.040) but not in comparison with the autologous group. Renal histology revealed glomerular and tubular damage in all groups. Signs of pathological hyperfiltration and microvascular injury were only observed in the human RBC group. In conclusion, perfusion of porcine kidneys with RBCs of different origin proved technically feasible. However, laboratory analysis and histology revealed more damage in the human RBC group compared to the other two groups. These results indicate that the use of allogeneic RBCs is preferable to human RBCs in a situation where autologous RBCs cannot be used for NMP.

## Introduction

In pre-clinical renal transplantation research, porcine kidneys are often used as the anatomy and physiology of the urinary system, as well as most tissue characteristics of pigs show many similarities with those of humans [1–3]. In addition, organ development and the pattern of disease progression make pigs an ideal model to study consequences of ischaemia-reperfusion injury (IRI) for translation to the human setting. Large animal models also allow for a realistic estimate regarding the quality and cost-effectiveness of new therapies [4].

A porcine autotransplantation model permits research of novel approaches related to kidney regeneration and repair without the interference of an allogeneic immune response [5,6]. A typical model should incorporate clinical donor conditions mimicking human surgery, kidney preservation using machine perfusion and functional recovery after transplantation. The need for oxygenation during pre-transplant (sub)normothermic (20–37°C) ex vivo machine perfusion (NMP) has been validated making an oxygen carrier essential in the NMP perfusate [7]. A leukocyte depleted red blood cell (RBC)-based solution is suitable for the perfusion of an isolated kidney under normothermic temperatures [8]. However, a large amount of porcine whole blood needs to be obtained to compose an RBC-based ex vivo perfusion solution with a near-physiological haematocrit. As the laboratory pig needs to survive both kidney donation and autotransplantation, this amount of blood cannot be retrieved from the same animal, especially since pigs are slightly anaemic by nature [9]. Therefore, an alternative is required in the form of RBCs from an allogeneic porcine or human source. An advantage of the use of human RBCs is that they are readily available in a hospital setting.

In contrast to the rather straightforward human blood group systems, pigs have no less than sixteen recognised blood group systems. Of these sixteen systems, the A-O system is the most important [10–12]. Data on the perfusion of pig kidneys with other-than-autologous sources of blood are scarce as the only setting in which this is possibly required is an autotransplant or xenotransplant model [4,10]. In the few studies that have been conducted, haemolytic transfusion reactions do not seem to occur in pigs that had not undergone transfusion previously [10]. However, it has been reported that the use of A-O incompatible whole-blood transfusions in pigs undergoing liver transplantation resulted in pulmonary hypertension and alterations in their coagulation response, eventually leading to death [13,14].

Most often, pre-clinical ex vivo perfusion experiments are carried out with *whole* blood. However, in the human clinical setting an NMP perfusate would never be composed of whole blood, as human whole blood is not typically available. Moreover, the plasma and buffy coat components of whole blood, which contain antibodies, clotting factors, activated leukocytes and thrombocytes, could be detrimental for ex vivo perfused organs. A more clinically relevant pre-clinical NMP protocol would therefore utilise a plasma free and leukocyte/thrombocyte depleted RBC-based perfusate. Perfusions of isolated porcine kidneys with such a solution, containing either allogeneic or human erythrocytes in the absence or diminished presence of an immune response have not been reported earlier. Aim of the present study was to determine whether an isolated porcine kidney can also be perfused ex vivo with a perfusion solution that is supplemented with allogeneic porcine or human RBCs instead of autologous RBCs as oxygen carriers.

## Materials and methods

### Kidney and blood retrieval

Three experimental groups (n = 5 each) were defined and kidneys were randomly distributed in this porcine model of renal donation after circulatory death (DCD). Viable porcine kidneys (sow; type: Topigs 20) as well as 2.5 litres of either autologous or allogeneic whole blood to which heparin (5000 international units per ml (IU), LEO® pharma, Ballerup, Denmark) was added to prevent blood from clotting, were obtained from a local slaughterhouse (Kroon Vlees, Groningen, the Netherlands). Human packed red blood cells (PRBC), type O positive, were provided by our hospital’s transfusion laboratory. Kidneys were exposed to 20 minutes of warm ischaemia between circulatory arrest and ex vivo cold flush with 180 mL of sodium chloride (0.9%) (Baxter, Utrecht, the Netherlands). Subsequently, kidneys were connected to a Kidney Assist Transport device (Organ Assist, Groningen, the Netherlands) for pulsatile oxygenated hypothermic machine perfusion (HMP) with University of Wisconsin Machine Perfusion Solution (Belzer UW-MPS, Bridge to Life Ltd, Columbia, SC) for 1.5–2.5 hours at 0–4°C. A leukocyte filter (BioR 02 plus BS PF, Fresenius Kabi, Zeist, the Netherlands) was utilised for leukocyte depletion in autologous and allogeneic porcine blood. This leucocyte depleted blood was centrifuged to obtain pure RBCs, without supernatant plasma and buffy coat. Next, porcine and human RBCs were washed with phosphate-buffered saline (PBS) and subsequently centrifuged once more to remove remaining plasma and PBS.

### Normothermic machine perfusion setup

The perfusion circuit consisted of a LifePort^®^ organ chamber with SealRing cannula (Organ Recovery Systems, Itasca, IL, USA), a magnetic pump head connected to a centrifugal pump unit (Deltastream DP2, Medos Medizintechnik AG, Stolberg, Germany) and an oxygenator with integrated heat exchanger (Hilite 800 LT, Medos Medizintechnik AG, Stolberg, Germany). The perfusate was oxygenated with 0.5 l/min carbogen (95% O_2_ / 5% CO_2_) and perfusate temperature was kept between 36 and 37°C. Pressure was measured directly after the SealRing cannula using a clinical-grade pressure transducer (TruWave disposable pressure transducer, Edwards Lifesciences, Irvine, CA, USA). This pressure sensor was zero-calibrated to the perfusion solution. Flow was monitored using an ultrasonic clamp-on flow probe (Transonic Systems Inc., Ithaca, NY, USA). All components of the NMP setup were controlled by a custom-made electronic interface and custom software (LabView Software, National Instruments Netherlands B.V., Woerden, the Netherlands).

The perfusion medium, with a final haematocrit between 0.35 and 0.45, consisted of 500 ml Williams’ Medium E (Gibco® William’s Medium E + GlutaMAX™, Life Technologies Limited, Bleiswijk, Netherlands) supplemented with amoxicillin-clavulanate 1000mg/200mg (Sandoz B.V., Almere, Netherlands), 40 g of albumin (Bovine Serum Albumin fraction V, Roche, Mannheim, Germany), 1000μmol/L creatinine (Sigma-Aldrich, Zwijndrecht, the Netherlands) and 350 ml pure red blood cells (RBCs). The kidney was perfused in a pressure controlled, pulsatile (60 beats per minute) sinusoid fashion at an arterial pressure of 110/70 mmHg during 7 hours.

### Urine and perfusate analysis

Hourly, arterial perfusate and urine samples were taken. Arterial blood gas samples of the perfusate were analysed after 0, 240 and 420 minutes of NMP. Perfusion parameters such as arterial flow rate and urine production were documented every 30 minutes. Concentrations of lactate dehydrogenase (LDH), aspartate aminotransferase (ASAT), urea, haemolysis index (H-index, a semi-quantitative measurement of the concentration of free haemoglobin in mg/dl), sodium, potassium, creatinine and albumin were measured with standard clinical assays. Creatinine clearance and fractional sodium excretion (FENa^+^) were calculated to determine renal function during NMP. Neutrophil gelatinase-associated lipocalin (NGAL) (NGAL pig ELISA kit, Enzo Life Sciences, Zandhoven, Belgium) levels were measured in the urine samples. Thiobarbituric acid reactive substances (TBARS) (Lipid Peroxidation (MDA) Assay Kit, Sigma-Aldrich B.V., Zwijndrecht, Netherlands), were measured in perfusate samples to quantify oxidative stress.

### Histology

Hollow needle biopsies were taken before the start of NMP and after 4 hours of NMP. At the end of each perfusion, larger surgical tissue samples from the renal cortex and medulla were collected. Formalin fixed paraffin embedded biopsies of the upper, lateral and lower renal cortex and medulla (t = -10; t = 240 and t = 420) were stained with haematoxylin and eosin (HE) to assess changes in tissue morphology. The biopsies were examined and a damage scoring system was developed, based on previously reported scoring systems [15], and validated by an experienced renal pathologist. As differences in histology between upper, lateral and lower pole were very minimal, per experiment three cortical biopsies of only the upper pole at t = -10, t = 240 and t = 420 were scored, by two independent blinded examiners. The sections were scored on a scale of 0–3 on glomerular dilatation, tubular dilatation and tubular necrosis (0 = none; 1 = mild; 2 = moderate; 3 = severe). In case of interobserver disagreement on the score, the two independent observers discussed their observations and reached a consensus.

### Statistical analysis

For all continuous longitudinally measured variables the area under the curve (AUC) was calculated. Unpaired one-sample *t*-tests were used to compare differences between two groups if the data were normally distributed an had homogeneity of variances. The Kruskal-Wallis test was utilised if data failed these assumptions. P-values of 0.05 or less were considered to indicate statistical significance. To indicate the level of significance in the graphs, asterisks were plotted in the graphs. One asterisk (*) indicates a level of significance of p ≤ 0.05. Two asterisks (**) indicate a p value of ≤ 0.01 and three asterisks (***) a p value of p ≤ 0.001.

## Results

### Kidney retrieval

Warm ischaemia time was between 20 and 22 minutes in all kidneys. Cold ischaemia times were not significantly different between groups: 29.6 ± 10.9 minutes in the autologous RBC group, 26.8 ± 10.26 minutes in the allogeneic RBC group and 32.0 ± 1.58 minutes in the human RBC group. There were no differences in HMP dynamics between groups and total HMP time did not differ significantly between groups either (129.5 ± 20.34 minutes in the autologous group; 124.2 ± 12.28 minutes in the allogeneic RBC group and 121.8 ± 28.07 minutes in the human RBC group).

### Perfusion dynamics

**Fig 1:**
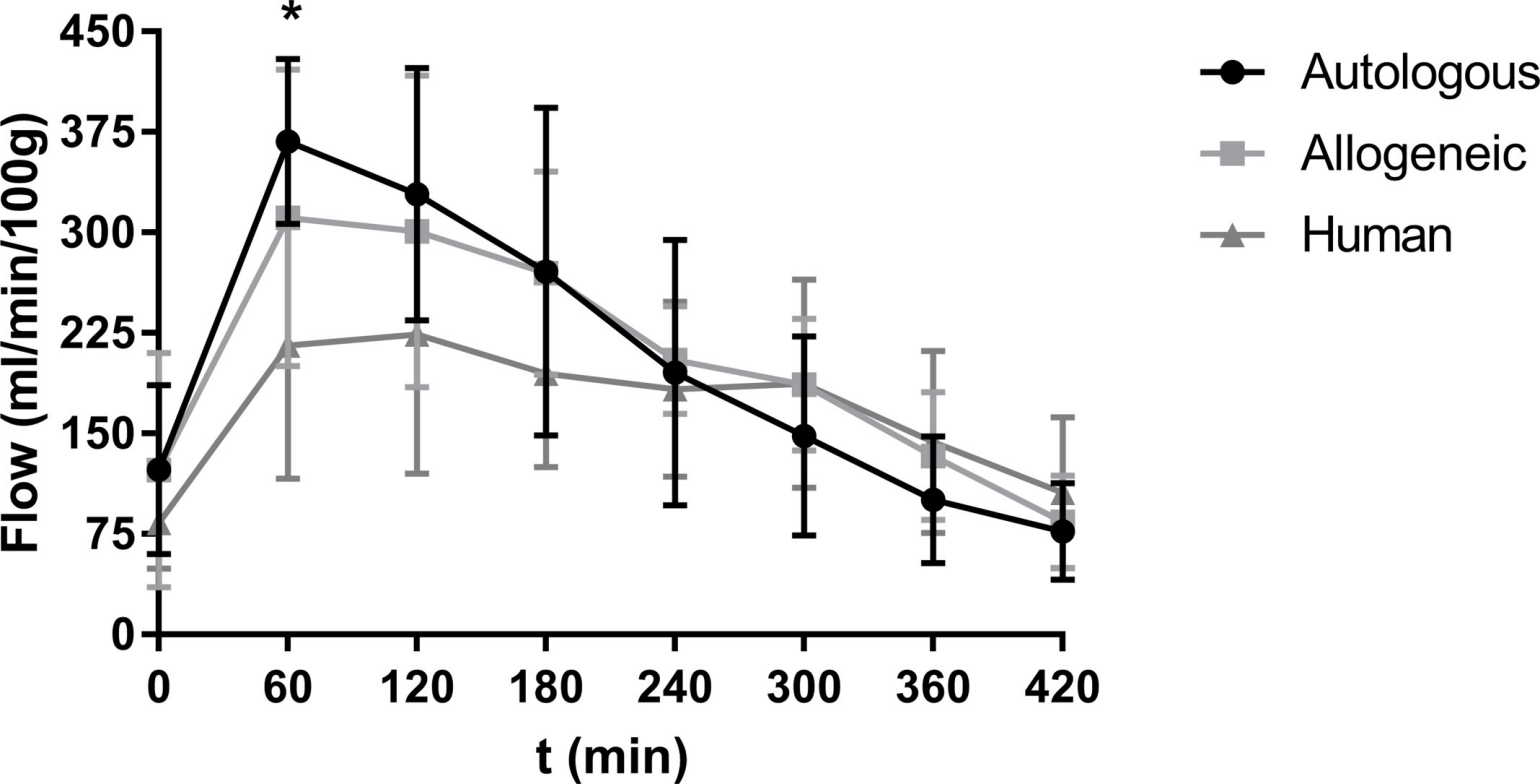
Mean flow per 100 g of the three experimental groups during NMP (± SD). * indicates statistical significance of ≤ 0.05.

Normothermic perfusion flow values showed a similar course in all groups. The flow rate increased during the first hour and decreased to 200–250 ml/min after 7 hours of NMP (Fig 1). Flow rates typically peaked at 1 hour after the start of NMP, with values that were highest in kidneys perfused with autologous RBCs and lowest in organs perfused with human RBCs. The peak flow value per 100 g after 60 minutes of perfusion was significantly higher in the autologous RBC group in comparison with the human RBC group (P = 0.0196) but not in comparison with the allogeneic RBC group. There was no significant difference in peak flow rate between the allogeneic and human RBC group. The pH values were between 7.12 and 7.38 in all experiments. The average weight gain during NMP was 58 ± 26% in the autologous group, 45 ± 37% in the allogeneic RBC group and 61 ± 30% in the human RBC group and there were no significant differences between groups. Macroscopic appearance of the kidneys did not differ between groups.

### Urine and perfusate analysis

Individual data on kidney function and damage markers of each kidney can be found in Table 1.

**Table 1:** Individual data per kidney per group at t = 60, t = 180, t = 300 and t = 420.

RBC type	Time	Serum creatinine (umol/l)	Creatinine clearance (ml/min)	FENa (%)	Albuminuria (mg/l)	NGAL (ng)	LDH (mmol/l)	ASAT (mmol/l)	BUN
Autologous 1	60	390	0.66	24	<3	^+^	247	42	1.8
	180	377	1.25	3	<3	^+^	469	98	2.2
	300	350	1.10	41	<3	^+^	615	165	2.5
	420	375	0.40	96	<3	^+^	631	211	3.0
Autologous 2	60	395	3.23	17	<3	11.52	246	57	2.1
	180	245	2.81	10	<3	2.26	420	79	2.2
	300	215	1.51	36	<3	1.60	535	99	2.7
	420	181	2.91	40	<3	5.69	510	128	3.1
Autologous 3	60	474	1.05	92	<3	9.66	223	51	2.3
	180	416	1.38	45	<3	3.14	348	134	2.7
	300	382	0.58	91	<3	3.56	437	236	3.0
	420	353	0.37	90	<3	2.22	501	380	3.3
Autologous 4	60	402	1.57	25	<3	4.43	567	43	1.6
	180	227	4.30	2	<3	2.69	642	61	1.8
	300	175	1.71	28	<3	1.74	666	71	2.0
	420	172	2.03	42	<3	3.31	618	73	2.3
Autologous 5	60	372	1.41	52	<3	7.88	265	69	2.2
	180	333	0.72	25	<3	1.95	459	111	2.5
	300	250	1.95	61	<3	2.19	531	129	2.9
	420	248	2.12	81	<3	6.64	595	166	3.2
Allogeneic 1	60	498	1.18	22	<3	1.54	268	47	2.3
	180	446	1.01	90	<3	4.21	363	78	2.5
	300	410	0.59	104	<3	1.66	379	125	2.7
	420	372	0.70	93	<3	2.07	385	185	3.1
Allogeneic 2	60	476	0.63	76	<3	2.97	250	52	2.2
	180	448	0.20	42	<3	1.76	381	90	2.5
	300	431	0.24	39	<3	2.11	488	135	2.8
	420	419	0.14	60	<3	1.38	543	183	3.0
Allogeneic 3	60	465	0.16	50	<3	0.48	164	29	1.8
	180	463	0.22	35	<3	0.76	272	46	2.4
	300	440	-	-	-	-	334	66	2.6
	420	442	0.13	88	<3	0.99	382	85	3.0
Allogeneic 4	60	505	1.19	27	<3	2.48	229	52	2.5
	180	438	0.91	25	<3	5.49	380	73	2.7
	300	364	1.74	33	<3	5.05	472	115	2.7
	420	314	1.59	49	<3	4.20	482	151	3.0
Allogeneic 5	60	494	1.40	53	<3	7.18	172	34	1.9
	180	422	1.21	6	<3	3.78	295	67	2.2
	300	341	1.39	13	<3	3.92	367	96	2.4
	420	376	0.22	51	<3	2.54	362	119	2.9
Human 1	60	446	1.65	63	38	1.39	289	46	1.6
	180	395	1.39	25	13	1.54	311	89	2.0
	300	272	3.31	49	15	3.02	569	203	2.3
	420	196	2.50	60	23	5.10	698	400	2.2
Human 2	60	431	1.82	67	63	0.94	378	46	1.7
	180	377	0.63	18	11	1.11	362	65	2.6
	300	331	0.88	29	11	2.45	436	191	2.8
	420	296	0.73	44	14	2.26	548	391	3.4
Human 3	60	289	6.92	20	20	4.10	254	45	1.6
	180	162	9.36	27	18	5.69	548	131	1.7
	300	112	3.42	49	30	3.80	843	545	2.0
	420	88	33.24	3	37	3.63	955	1157	2.0
Human 4	60	267	23.77	5	18	^+^	254	40	1.2
	180	112	5.71	3	17	1.66	326	55	1.4
	300	59	9.18	8	30	3.19	424	78	1.6
	420	45	2.30	43	40	3.47	412	93	1.8
Human 5	60	564	1.29	114	61	3.05	342	90	2.3
	180	459	0.65	90	44	2.20	523	178	2.2
	300	380	0.96	90	37	3.69	621	371	1.8
	420	350	0.45	116	33	2.24	786	648	1.5

RBC, red blood cell; FENa, fractional excretion of sodium; NGAL, neutrophil gelatinase-associated lipocalin; LDH, lactate dehydrogenase; ASAT, asparatate aminotransferase; BUN, blood urea nitrogen.

^+^no urine sample left for additional NGAL analysis.

- kidney did not produce urine at this time point.

**Fig 2:**
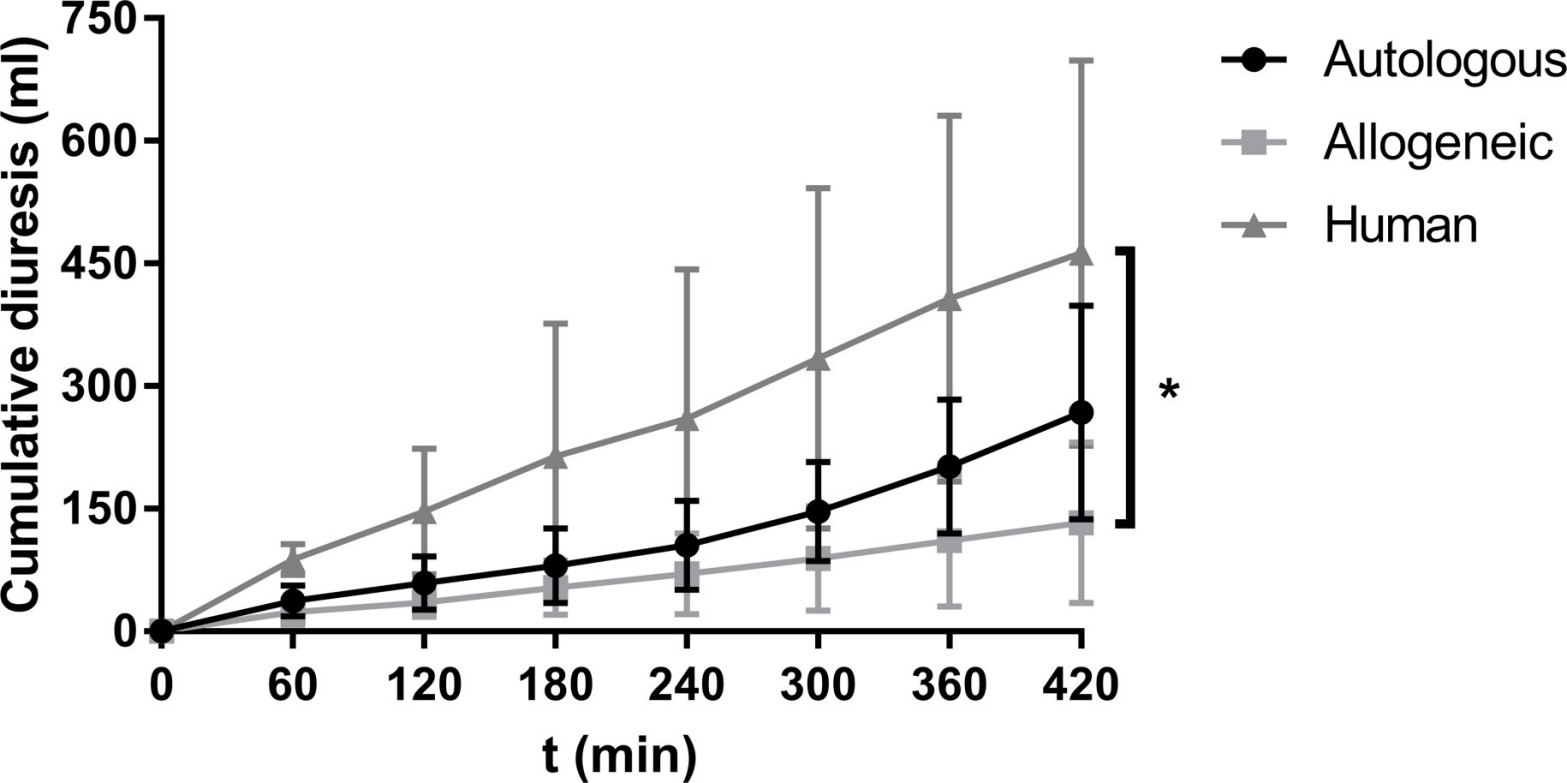
Mean cumulative diuresis (ml) of the three experimental groups during NMP (± SD). * indicates statistical significance of ≤ 0.05.

Highest levels of cumulative diuresis were seen in the human RBC group (Fig 2). Urine output in the human RBC group was significantly higher than in the allogeneic RBC group (P = 0.010). The human RBC group and the autologous RBC group as well as the allogeneic RBC group and the autologous RBC group did not differ significantly.

**Fig 3:**
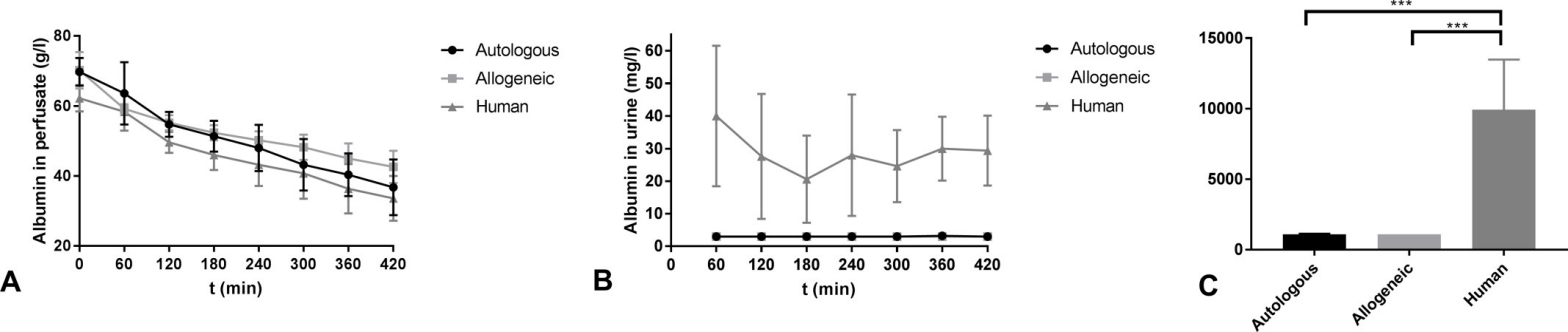
Albumin levels. (A) Albumin concentration (g/l) in the perfusate and (B) albumin concentration in urine (g/l) and (C) AUC of albumin in urine of the three experimental groups during NMP (mean ± SD). *** indicates statistical significance of ≤ 0.001.

FENa^+^ levels were very high in each group, indicating that tubular function was severely impaired during NMP. FENa^+^ in the autologous group (69.48 ± 26.81% at t = 420 min) was not significantly different from the allogeneic RBC group (68.18 ± 21.04% at t = 420 min) or from the human RBC group (53.18 ± 40.97% at t = 420 min). The allogeneic RBC group did not show statistically significant different values compared to the human RBC group. Creatinine clearance in the human RBC group (7.848 ± 14.230 ml/min at t = 420 min) showed higher values compared to the autologous RBC group (1.567 ± 1.132 ml/min at t = 420 min), although this difference did not reach statistical significance. However, end-perfusion values at t = 420 min in the human RBC group were significantly higher when compared to the allogeneic RBC group (0.5567 ± 0.624 ml/min, P = 0.049). The kidneys in the human RBC group leaked large amounts of albumin into the urine, leading to decreased levels of albumin in the perfusate (Fig 3A). However, the concentration of albumin in the perfusate dropped discrepantly fast in all groups in comparison with the amount secreted in the urine. The levels of urinary albumin (Fig 3B and 3C) in the human RBC group were significantly higher than in the allogeneic RBC group (P < 0.001) and the autologous RBC group (P < 0.001).

**Fig 4:**
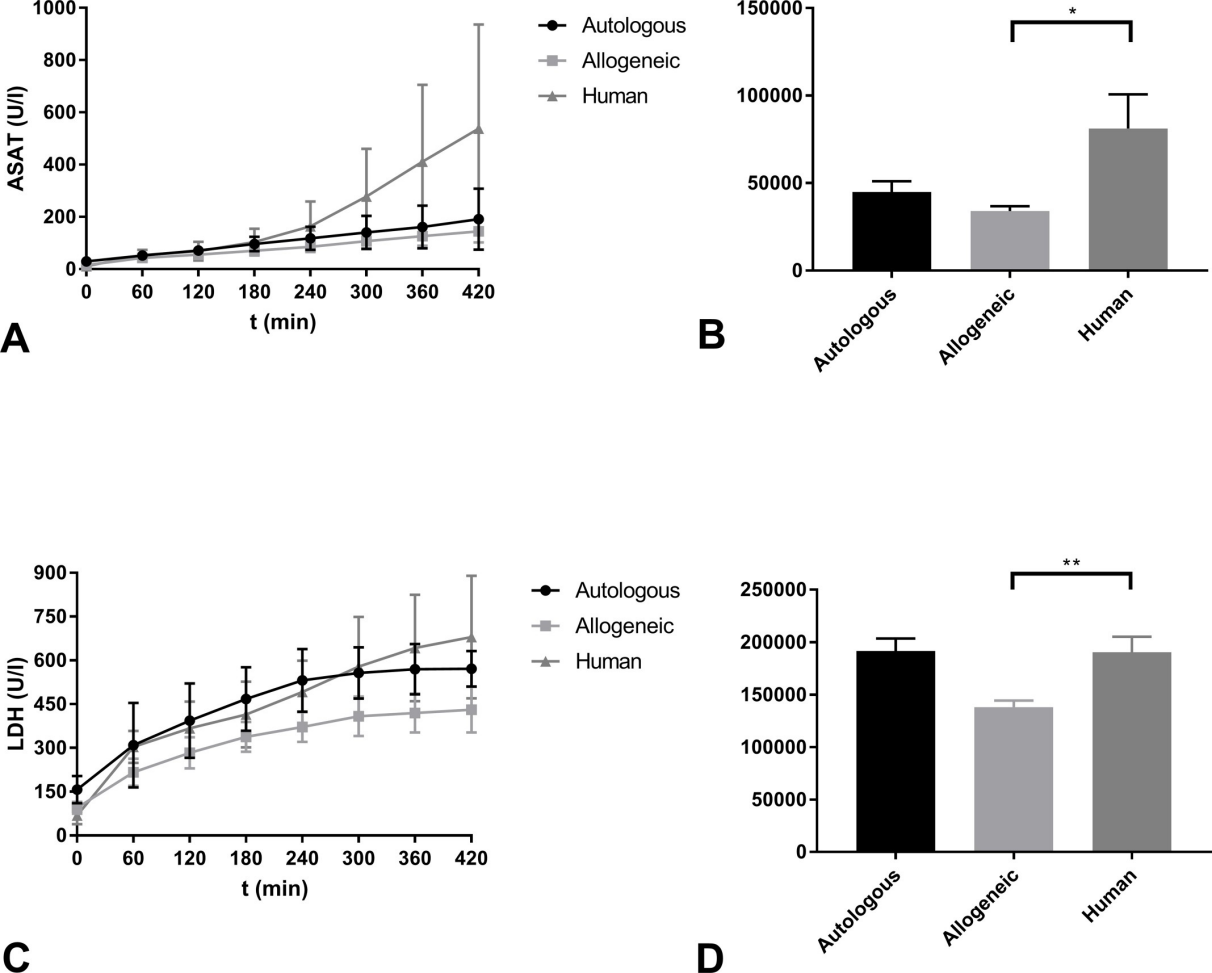
Markers of general cell injury. (A) ASAT levels in perfusate (U/l) and (B) AUC of the three experimental groups during NMP (mean ± SD). (C) LDH levels in perfusate (U/l) and (D) AUC of the three experimental groups during NMP (mean ± SD). * indicates statistical significance of ≤ 0.05. ** indicates statistical significance of ≤ 0.01.

ASAT and LDH were measured as markers of general renal cell injury. ASAT levels in the human RBC group were significantly higher than in the allogeneic RBC group (P = 0.040) (Fig 4A and 4B). There were no other significant differences between groups. Release of LDH into the perfusate was also significantly higher in the human RBC group in comparison with the allogeneic group (P = 0.019). There were no significant differences in LDH levels between the autologous RBC group and the human RBC group (Fig 4C and 4D).

**Fig 5:**
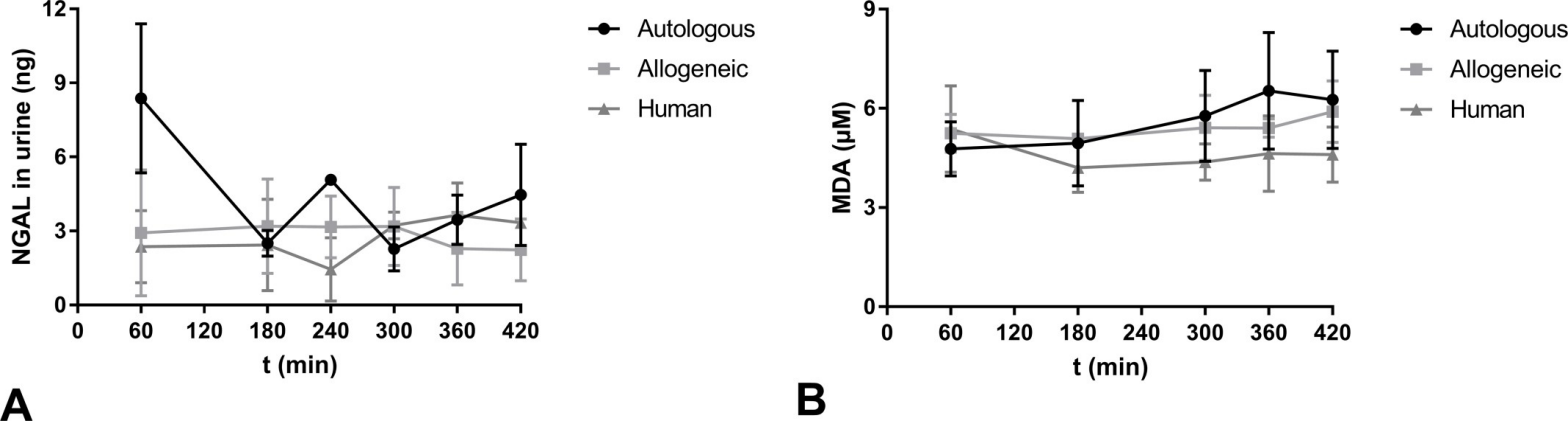
NGAL and TBARS quantification. (A) Absolute NGAL (ng) levels in urine and (B) TBARS (MDA in μM) in the perfusate of the three experimental groups during NMP (mean ± SD).

Absolute NGAL levels in the urine were comparable in the allogeneic and human RBC group. Levels at the start of perfusion were highest in the autologous group although this did not reach statistical significance (Fig 5A). End perfusion levels at t = 420 were similar between groups and did not differ significantly with mean values of 4.464 ± 2.047 ng in the autologous group, 2.236 ± 1.25 ng in the allogeneic group and 3.339 ± 1.178 ng in the human group. TBARS, quantified by measurement of MDA were similar in all groups and there were no significant differences (Fig 5B). Mean end perfusion values at t = 420 measured 6.256 ± 1.475 μM in the autologous group, 5.902 ± 0.934 μM in the allogeneic group and 4.600 ± 0.835 μM in the human group. Measurement of free haemoglobin in serum was used as a marker for haemolysis during reperfusion. The haemolysis index (H-index) showed a moderate increase over time but remained in the range of slightly haemolytic, as levels over 100 were not reached. H-indices were not significantly different between groups.

### Histology

Protein deposition and widening of Bowman’s capsules was observed in renal glomeruli after NMP with autologous RBCs, indicating a state of glomerular hyperfiltration. RBCs were present in tubules, leading to haematuria. In addition, tubular epithelial denudation and cellular necrosis were seen in proximal tubules. Denuded and necrotic cells fall into the tubular lumen and obstruct the tubule in the form of proteinaceous casts. In the lumen of distal tubules intratubular cell detachment was seen.

**Fig 6:**
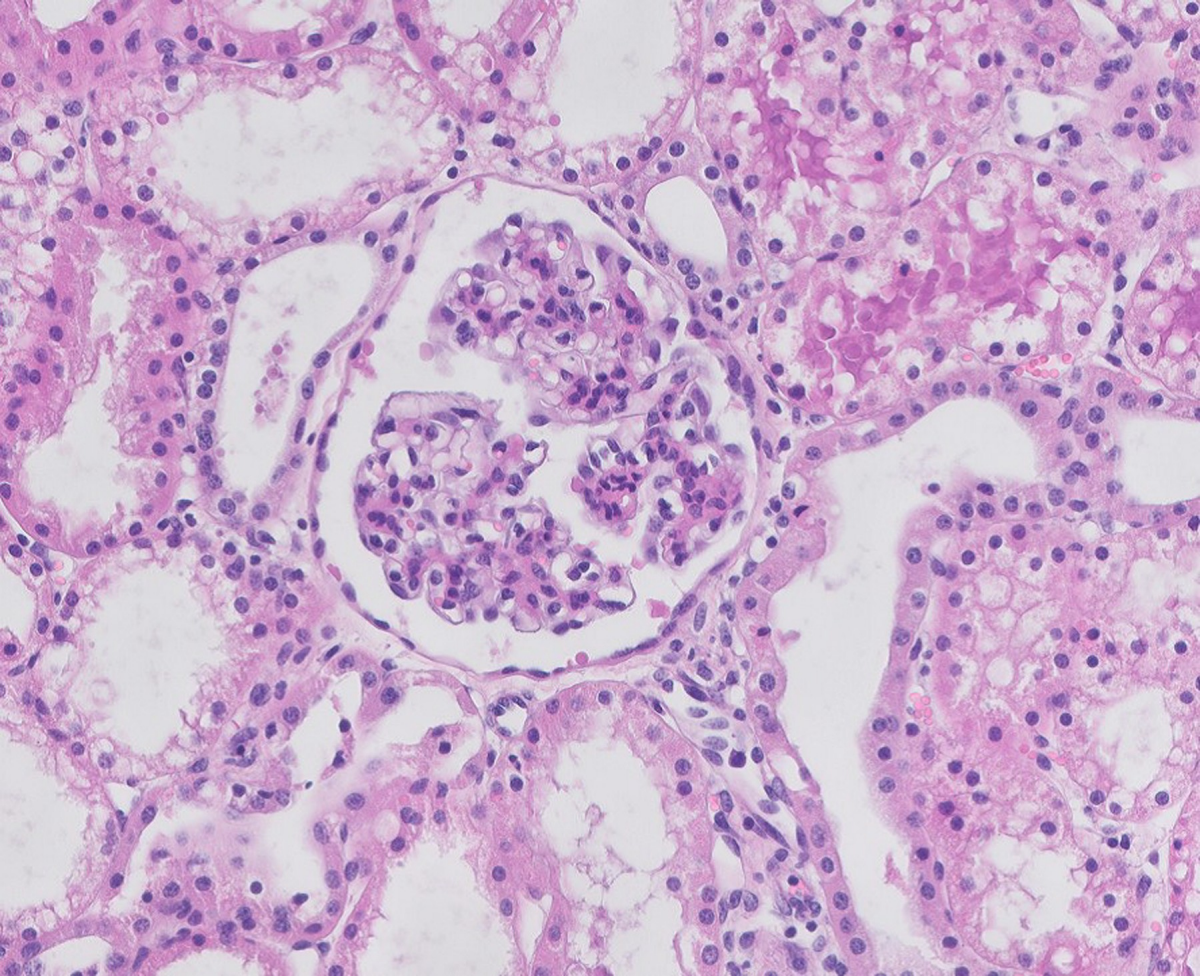
Histological evaluation if a kidney perfused with allogeneic RBCs. An example of a light microscopy image of HE-stained biopsy of a biopsy of a porcine kidney perfused with allogeneic RBC-based solution after t = 420 min of NMP.

In the allogeneic RBC group the same extent of typical ischaemia/reperfusion-associated damage was seen; Bowman’s capsules were damaged, brush borders were no longer intact and tubular lumens were dilated (Fig 6).

**Fig 7:**
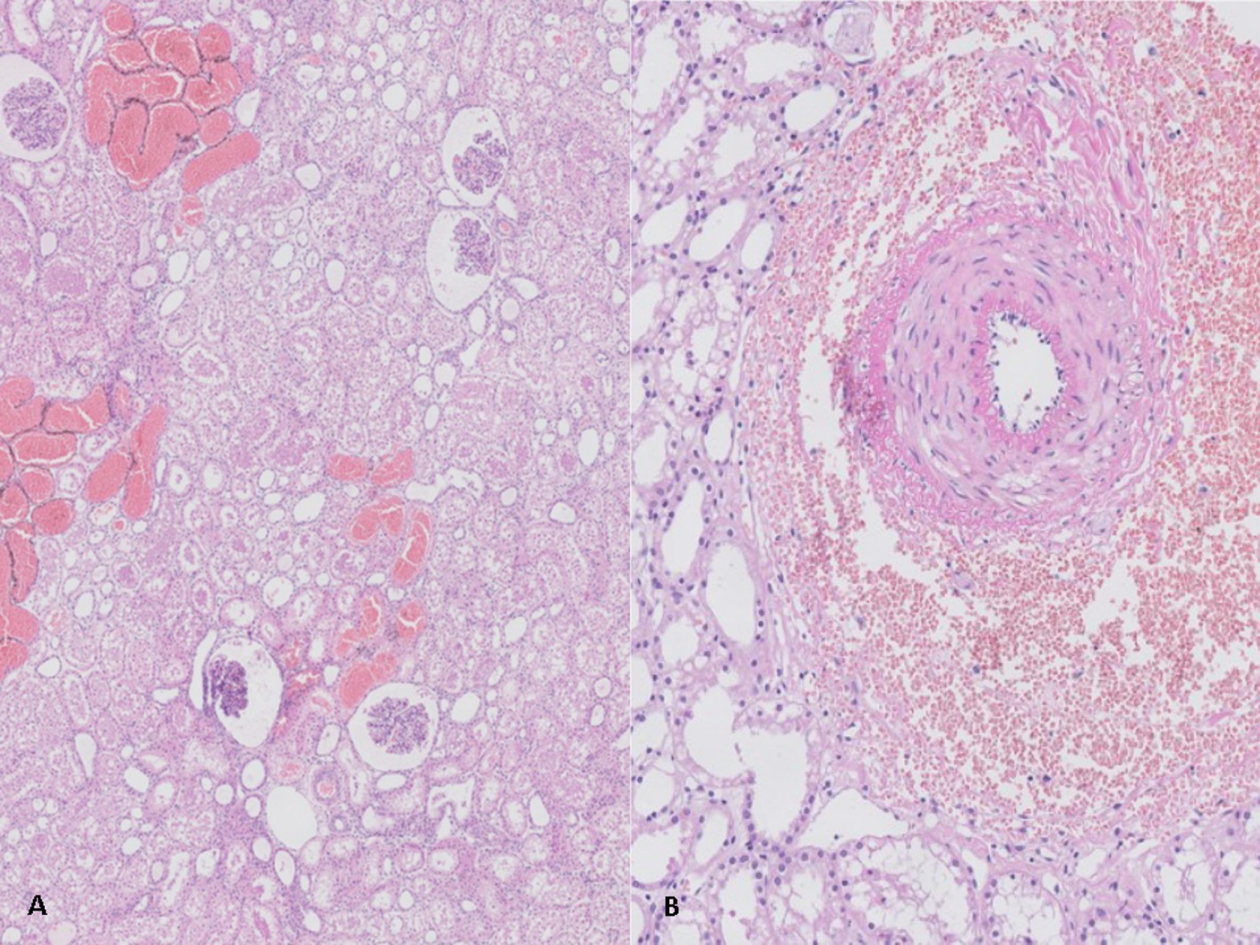
Histological evaluation of a kidney perfused with human RBCs. (A) An example of a light microscopy image of HE-stained biopsy of a biopsy of a porcine kidney perfused with human RBC-based solution after t = 420 min of NMP; (B) Focal lesion around a blood vessel in a HE-stained biopsy of a porcine kidney perfused with a human RBC-based solution after 420 minutes of NMP.

In addition to injury seen in the porcine RBC groups, a more serious state of glomerular hyperfiltration occurred during NMP with human RBCs; with corresponding lesions in Bowman’s capsule (Fig 7A). Extensive dilatation of tubular lumens and signs of pyknosis were observed. More detailed views at high magnification showed degeneration of tubular epithelial cells in which nuclei shrank whilst undergoing apoptosis. Tubular vacuolisation was also seen in this human RBC group, which along with the extensive tubular dilatation, could result in tubular basement membrane rupture. A focal lesion, with RBCs around a blood vessel, was noted in two postperfusion biopsies from different experiments in the human RBC group, indicating massive microvascular injury (Fig 7B).

**Fig 8:**
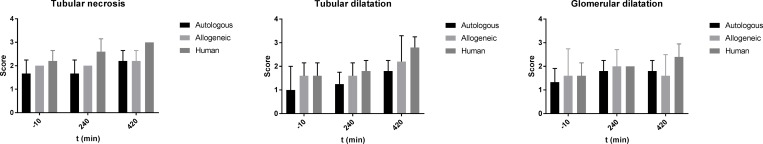
Histological damage scoring of cortical biopsies taken at t = -10, t = 240 and t = 420 (mean ± SD).

Of each experiment three cortical biopsies, taken after prior to the start (t = -10), after 240 minutes and after 420 minutes of NMP, were scored on a scale of 0–3 on glomerular dilatation, tubular dilatation and tubular necrosis (Fig 8). There were no significant differences in those scores between groups, although there did seem to be a trend towards higher injury scores on all three subscales in the human RBC group.

## Discussion

This study compared renal function and structural cell injury in isolated porcine kidneys during normothermic machine perfusion with autologous, allogeneic porcine, or human RBCs. The NMP setup was used in conjunction with a porcine model of donation after circulatory death (DCD) kidney donation, in which viable slaughterhouse-derived kidneys were used. We observed that kidneys in our study showed ischaemic damage similar to that seen in DCD models in pigs [16,17]. Hence, we feel that this laboratory-animal saving porcine slaughterhouse model had a good clinical translation relevance. To our knowledge this is the first study in which ischaemically damaged porcine kidneys were ex vivo perfused under normothermic conditions with an allogeneic porcine or human RBC-based perfusion solution, which is also more clinically relevant than experimental NMP models which utilise whole blood as a perfusion solution.

Peak flow values at t = 60 were significantly higher in the autologous group in comparison with the human group but not in comparison with the allogeneic group. The exact cause of this difference remains unclear. From unpublished data from our group in which we perfused kidneys with perfusion solutions with different compositions we can conclude that the composition of the solution is of vital importance and that in a well-balanced solution peak flow values are seen after approximately 60 minutes. Thereafter, the kidneys seemed to be able to regulate the microvasculature. In those series of experiments, kidneys in which this peak flow did not occur performed inferiorly in comparison with the other groups. Therefore, we hypothesise that the peak flow at t = 60 in the autologous and allogeneic, and the absence of that in the human group, could be the result of the composition of the solution.

Elevated FENa^+^ levels stood out in all experimental groups, indicating that kidney function was severely impaired, as expected in this DCD model. The raised values likely result from acute tubular necrosis (ATN), and are similar to FENa^+^ values reported in other renal NMP studies [1,18]. Perfusion with human RBCs led to high levels of albuminuria. Normally, the glomerular capillary wall functions as a filter that allows passage of small molecules, but almost completely restricts the passage of molecules the size of albumin or larger. In glomerular proteinuric states, the filtration barrier between the blood and urinary space is damaged [19]. The significantly higher levels of albuminuria that we observed in the human RBC group are most probably the result of extensive, irreversible damage to the integrity of the glomerular membrane. This is not a likely sign of typical ischaemia-reperfusion injury, as such injury most often affects tubuli. Since these signs of glomerular membrane damage were not present in the other two experimental groups with a similar extent of IRI, we hypothesise that this serious glomerular injury could be a result of xeno-reactions compromising glomerular vascular wall integrity. The concentration of albumin in the perfusate dropped discrepantly fast in all groups in comparison with the amount secreted in the urine. The most likely explanation for this decrease in albumin levels is that albumin adheres to the plastic tubing used in the NMP circuit in large quantities, causing it to functionally disappear from the circulating perfusate. Inflammation as a result of ischaemia-reperfusion injury could be an additional explanation for the reduction in albumin perfusate levels. Although we utilised a leukocyte depleted perfusate, tissue resident leukocytes may still have triggered such an inflammatory response during NMP, which could be even more pronounced in the xeno-setting that characterised our human RBC group. In response to injury, acute-phase proteins, e.g. albumin, decrease during inflammation to save amino acids and energy for the synthesis of other proteins [20].

The human RBC group showed higher values of cumulative diuresis and creatinine clearance in comparison with the other two groups. Since levels of albuminuria were also highest in this group, these findings could be explained by an osmotic effect as a result of the loss of albumin in the urine. Since most probably glomerular membranes were critically damaged in kidneys perfused with human RBCs, this in turn resulted in a lower ultrafiltration-inhibitory colloid osmotic glomerular membrane gradient as albumin was excreted into the urine. This below normal colloid osmotic pressure likely allowed for more fluid to pass through to the tubules and thus resulted in more diuresis.

Renal injury markers such as ASAT and LDH were significantly higher in kidneys that were perfused with a human RBC-based solution. Both damaged kidney tissue and haemolysis could cause these higher values of ASAT and LDH. Although H-indices were not significantly different between groups, the highest levels of free haemoglobin were measured in the human RBC group indicating that more haemolysis may have occurred in this group than in the other groups. Haemolysis during perfusion causes an increase in vascular resistance and tissue oedema during prolonged periods of ex vivo machine preservation [21].

All biopsies revealed structural cell injury which might be explained by the fact that the experiment was based on a DCD model. Organs recovered from DCD donors are known to have sustained considerable IRI, as a result of inevitable warm ischaemia incurred between circulatory arrest in the donor and the start of systemic cooling during organ retrieval. The pathological condition of IRI results in numerous cellular injuries and disruption of cellular membranes, including cytoskeletal microtubules and mitochondrial membranes [22–25]. Widening of Bowman’s capsules and cellular necrosis of proximal tubular epithelial cells were observed after NMP with all types of RBCs. The pathogenic mechanism of IRI can lead to ATN, resulting in morphological changes to which the proximal tubules are most susceptible. Tubular epithelium is prone to IRI for two reasons: its high metabolic activity rates in parts of the nephrons where resorption and secretion occurs and its location after the glomerulus with only low perfusion flow from the vasa recta. The obstruction of tubular lumens by cellular debris combined with the leakage of solutes across injured tubular epithelium contributes to an increased hydrostatic pressure in Bowman’s capsule. This increased pressure will eventually lead to the widening of Bowman’s space. However, ATN is highly reversible and will most likely recover when renal ischaemia is corrected after reperfusion.

Allogeneic RBCs in this study were not cross-matched before use, thus not taking the porcine A-O and other blood group systems into account. Despite the potential random A-O mismatch, perfusion proved feasible and renal histological appearance was similar to that of kidneys perfused with autologous RBCs. In combination with our results on renal function and perfusate injury markers, it can be concluded that NMP with allogeneic porcine RBCs did not differ markedly from the control group with autologous porcine RBCs.

In the human RBC group histology revealed more extensive and most likely irreversible damage, with maximum scores in all three quantitative injury categories on nearly all the biopsies. The focal lesions centred around blood vessels, seen in two experiments, indicate massive microvascular injury possibly as a result of an xeno-reactive response of the porcine endothelium to human RBCs or a xeno-reaction as a result of small amounts of plasma or leukocytes that remained in the perfusion solution or were tissue resident. Prior studies have shown that human serum can lead to complement mediated activation of porcine endothelial cells, which is known to play a central role in the process of hyperacute rejection [26].

Our study had several limitations. First, the physiology of exsanguination of animals at a slaughterhouse is not completely equivalent to a donor with actual cardiac arrest. Also, animals at a slaughterhouse probably experience more acute stress than typical DCD donors and kidneys could be too damaged to be suitable for actual transplantation. Although NMP before transplantation appears to be beneficial, the optimal duration of NMP has not yet been determined. Longer perfusion times may be needed to recover function in ischaemically damaged kidneys [27]. However, the increasing levels of ASAT during our experiments suggest that the observed renal injury might partly be caused by the duration of NMP. The concentrations of ASAT showed a linear increase over time in the experimental groups with allogeneic and autologous RBCs. The LDH concentrations seemed to stabilise after approximately five hours of NMP with autologous and allogeneic RBCs. Understanding the exact implication of this finding is difficult, as other studies that have reported NMP of isolated porcine kidneys usually relied on a shorter duration of ex vivo perfusion [1]. In the experiments with human RBCs, ASAT and LDH release did not stabilise during the seven hours of perfusion, indicating more extensive renal cellular injury and/or haemolysis. As mentioned earlier, in this study allogeneic RBCs were not cross-matched before NMP therefore not taking the porcine blood group system into account, which can be considered as a significant limitation in this study. As blood typing is a relatively simple procedure it should be considered in future studies.

A possible alternative to RBCs is the use of artificial blood substitutes such as perfluorochemical (PFC) or a haemoglobin solution [28,29]. Two limitations of PFC are its insolubility in the aqueous phase and the necessity of high oxygen pressures to maximise oxygen carrying capacity [30]. Early haemoglobin solutions had several drawbacks in the clinical setting including renal impairment in patients who received multiple injections of this solution as a result of impurities. The next generation of haemoglobin solutions have addressed this impurity issue and several successful pre-clinical studies have been performed [31]. However, initial enthusiasm has been tempered as two major problems caused by haemoglobin solutions were identified: excessive systemic vasoconstriction and oxidative tissue damage [32].

The results of our experiments suggest that allogeneic porcine RBCs do not cause more renal damage than autologous RBCs, but a *human* RBC-based perfusion solution does lead to significantly more injury of isolated porcine kidneys during NMP. We conclude that, to enable NMP of porcine kidneys with an RBC-based perfusion solution in an autotransplantation model, allogeneic porcine RBCs are the best choice, as they do not seem to lead to unacceptable additional injury.

## Supporting information

S1 Data (PDF)
