# Supplementary material for: Normothermic machine perfusion of ischaemically damaged porcine kidneys with autologous, allogeneic porcine and human red blood cells
Source: PLoS One. 2020 Mar 10;15(3):e0229566. doi: 10.1371/journal.pone.0229566 (PMC7064242; doi:10.1371/journal.pone.0229566)
Supplement: S1 Data — (PDF) [file pone.0229566.s001.pdf]

				ASAT	Creat	Na	LDH	Albumin	MDA	uCreat	uNa	uAlbumin	uNGAL	
Experiment	Flow	Flow/100gr	t (min)	(U/L)	(umol/L)	(mmol/L)	(U/L)	(g/L)		(mmol/L)	(mmol/L)	(mg/L)	(ug/L)	Diuresis/h (ml)
**Autologous RBC**	554	180,23	**0**	33	94	149	135	67						0,0
	955	310,68	**60**	42	390	149	247	76	4,96	1,4	129	3		11,0
	670	217,96	**120**	64	391	154	360	60		4,0	74	3		4,5
	446	145,09	**180**	98	377	157	469	58	5,35	4,7	58	3		6,0
	330	107,36	**240**	137	373	164	568	58		2,6	102	3		8,0
	231	75,15	**300**	165	350	161	615	54	7,56	0,8	151	3		29,0
	125	40,66	**360**	183	358	163	613	49	9,31	0,5	162	3		27,0
	78	25,37	**420**	211	375	163	631	48	8,55	0,4	166	3		22,5
**Autologous RBC**	380	133,39	**0**	36	643	144	119	72						0,0
	1000	351,04	**60**	57	395	150	246	68	5,27	1,8	118	3	271	42,5
	800	280,83	**120**	67	281	158	319	55		2,2	53	3		52,5
	420	147,44	**180**	79	245	161	420	46	5,59	1,5	102	3	82	27,5
	250	87,76	**240**	83	233	166	441	41		0,8	143	3		25,0
	215	75,47	**300**	99	215	167	535	38	5,93	0,5	139	3	41	39,0
	185	64,94	**360**	104	198	169	483	35	6,74	0,4	147	4	62	50,0
	165	57,92	**420**	128	181	170	510	34	6,55	0,4	150	3	72	79,0
**Autologous RBC**	153	57,13	**0**	25	630	149	185	75						0,0
	1070	399,57	**60**	51	474	149	223	63	5,65	0,5	144	3	161	60,0
	994	371,19	**120**	86	436	152	308	54		1,0	127	3		27,5
	920	343,55	**180**	134	416	156	348	52	6,55	0,8	135	3	73	43,0
	655	244,59	**240**	174	388	158	407	49		0,6	148	3	145	35,0
	490	182,98	**300**	236	382	161	437	43	6,01	0,4	154	3	108	33,0
	290	108,29	**360**	285	359	159	468	41	6,60	0,4	159	3	98	27,0
	222	82,90	**420**	380	353	159	501	37	5,87	0,4	162	3	114	19,5
**Autologous RBC**	523	186,61	**0**	21	624	145	226	70						0,0
	1287	459,22	**60**	43	402	152	567	58	4,48	1,4	131	3	164	27,0
	1305	465,64	**120**	52	290	158	618	55		3,6	76	3		18,0
	1182	421,75	**180**	61	227	160	642	52	3,63	3,9	66	3	179	15,0

	916	326,84	**240**	64	184	163	674	49		2,0	124	3		17,0
	699	249,41	**300**	71	175	165	666	46	5,67	0,6	158	3	58	30,0
	422	150,57	**360**	73	178	169	640	43	4,90	0,4	159	3	60	52,5
	313	111,68	**420**	73	172	167	618	39	5,78	0,4	161	3	63	52,5
**Autologous RBC**	200	58,36	**0**	34	712	145	122	65						0,0
	1095	319,50	**60**	69	372	150	265	53	3,52	0,7	146	3	175	45,0
	1055	307,83	**120**	87	347	153	361	50		1,7	136	3		8,0
	1019	297,33	**180**	111	333	158	459	49	3,62	1,1	130	3	150	13,0
	727	212,13	**240**	131	309	157	565	43		0,4	154	3		43,0
	545	159,02	**300**	129	250	162	531	35	3,71	0,4	159	3	30	73,0
	476	138,89	**360**	161	246	165	643	34	5,11	0,3	158	3	41	120,0
	370	107,96	**420**	166	248	162	595	26	4,53	0,2	162	3	42	158,0
**Allogeneic RBC**	385		**0**	19	707	150	112	68						0,0
	914		**60**	47	498	152	268	60	4,47	2,2	147	3	96	16,0
	830		**120**	59	472	152	321	55		0,5	155	3		35,0
	635		**180**	78	446	154	363	50	4,98	0,5	155	3	78	54,0
	445		**240**	100	441	159	378	47		0,4	158	3	58	45,0
	363		**300**	125	410	159	379	42	5,43	0,4	162	3	46	36,0
	295		**360**	157	391	163	390	38	5,22	0,4	161	3	52	44,0
	259		**420**	185	372	165	385	35	5,21	0,4	165	3	53	39,0
**Allogeneic RBC**	215	97,99	**0**	9	741	150	66	68						0,0
	647	294,90	**60**	52	476	150	250	57	5,46	0,6	144	3	99	30,0
	685	312,22	**120**	75	437	150	346	52		0,7	143	3		7,0
	511	232,91	**180**	90	448	153	381	50	5,24	0,9	129	3	293	6,0
	381	173,66	**240**	106	434	154	415	48		1,0	128	3	333	5,0
	345	157,25	**300**	135	431	157	488	48	6,74	0,9	128	3	302	7,0
	248	113,04	**360**	165	407	155	516	45	5,31	0,8	132	3	234	3,0
	125	56,97	**420**	183	419	155	543	44	6,62	0,6	133	3	230	6,0
**Allogeneic RBC**	505	252,25	**0**	19	615	144	116	77						0,0
	940	469,53	**60**	29	465	149	164	60	5,47	0,9	145	3	96	5,0

	898	448,55	**120**	38	457	151	232	58		0,9	143	3		5,0
	734	366,63	**180**	46	463	152	272	54	4,96	1,2	139	3	151	5,0
	420	209,79	**240**	54	452	153	304	53						0,0
	482	240,76	**300**	66	440	155	334	50	4,00					0,0
	373	186,31	**360**	76	429	155	359	49	5,22	0,7	152	3	162	10,0
	253	126,37	**420**	85	442	157	382	47	4,63	0,5	157	3	142	7,0
**Allogeneic RBC**	187	80,08	**0**	16	832	157	97	74						0,0
	617	264,21	**60**	52	505	154	229	60	5,73	1,8	146	3	124	20,0
	646	276,62	**120**	58	475	155	290	56		1,8	130	3		7,0
	673	288,19	**180**	73	438	158	380	54	4,97	1,5	135	3	343	16,0
	608	260,35	**240**	87	398	159	424	51		1,3	140	3	175	23,0
	499	213,68	**300**	115	364	163	472	50	5,19	1,0	147	3	133	38,0
	365	156,30	**360**	129	329	165	462	45	5,88	0,8	149	3	104	45,0
	229	98,06	**420**	151	314	164	482	42	6,30	0,6	153	3	84	50,0
**Allogeneic RBC**	178	61,19	**0**	12	787	154	84	64						0,0
	627	215,55	**60**	34	494	154	172	59	4,82	0,9	149	3	156	46,0
	485	166,74	**120**	46	468	155	225	55		3,2	106	3		5,0
	556	191,14	**180**	67	422	160	295	54	5,24	3,4	82	3	420	9,0
	513	176,36	**240**	81	363	163	337	52		3,3	85	3	396	11,0
	392	134,76	**300**	96	341	163	367	51	5,70	1,9	118	3	261	15,0
	226	77,70	**360**	103	357	165	372	48	5,38	1,1	142	3	265	8,0
	161	55,35	**420**	119	376	166	362	45	6,75	0,7	157	3	363	7,0
**Human RBC**	347	124,60	**0**	16	679	150	82	60						0,0
	590	211,85	**60**	46	446	156	289	57	3,70	0,7	155	38	22	63,0
	490	175,94	**120**	47	399	157	321	50		1,2	139	20		12,0
	665	238,78	**180**	89	395	168	311	53	3,43	1,2	127	13	56	27,5
	715	256,73	**240**	117	323	167	465	50		0,8	136	11	32	55,0
	698	250,63	**300**	203	272	168	569	48	4,04	0,5	150	15	28	108,0
	624	224,06	**360**	308	221	168	683	43	3,94	0,4	155	18	42	130,0
	490	175,94	**420**	400	196	169	698	38	3,63	0,3	156	23	52	98,0

**Human RBC**	100	39,96	**0**	8	733	148	90	59						0,0
	235	93,90	**60**	46	431	149	378	56	6,76	0,6	138	63	12	78,5
	228	91,11	**120**	47	376	151	345	47		1,1	103	40		20,0
	262	104,69	**180**	65	377	157	362	46	5,10	1,1	81	11	85	13,0
	374	149,44	**240**	106	355	157	407	46		1,0	93	11	96	10,0
	437	174,62	**300**	191	331	160	436	45	5,20	0,7	98	11	98	25,0
	386	154,24	**360**	283	307	161	514	43	6,43				81	30,0
	235	93,90	**420**	391	296	164	548	41	5,27	0,5	122	14	87	26,0
**Human RBC**	299	110,82	**0**	18	666	147	97	64						0,0
	680	252,04	**60**	45	289	154	254	56	5,78	1,2	126	20	41	100,0
	665	246,48	**120**	71	261	173	362	52		1,2	69	18		168,0
	386	143,07	**180**	131	162	176	548	43	4,51	0,4	118	18	25	227,5
	310	114,90	**240**	281	129	174	628	35		0,3	161	55		80,0
	265	98,22	**300**	545	112	174	843	32	3,87	0,2	151	30	33	115,0
	224	83,02	**360**	861	92	175	897	28	3,59	0,2	157	26	40	84,0
	241	89,33	**420**	1157	88	172	955	26	3,91	3,0	166	37	62	58,5
**Human RBC**	132	70,45	**0**	10	644	145	45	60						0,0
	672	358,67	**60**	40	267	151	254	68	4,33	3,4	99	18	x	112,0
	701	374,15	**120**	48	150	160	285	53		1,5	30	6		63,0
	518	276,47	**180**	55	112	163	326	46	3,43	1,2	60	17	52	32,0
	467	249,25	**240**	66	85	170	381	46		0,8	95	26		40,0
	528	281,81	**300**	78	59	174	424	45	4,10	0,5	118	30	49	65,0
	358	191,08	**360**	86	53	175	425	38	4,14	0,2	155	39	57	78,0
	267	142,51	**420**	93	45	174	412	35	4,63	0,1	166	40	56	62,0
**Human RBC**	150	70,54	**0**	5	815	151	29	68						0,0
	345	162,24	**60**	90	564	147	342	55	6,31	0,5	148	61	35	87,0
	495	232,78	**120**	131	479	149	522	46		0,5	143	54		30,0
	447	210,20	**180**	178	459	151	523	42	4,55	0,5	147	44	61	36,0
	310	145,78	**240**	250	425	151	577	39		0,5	138	37	64	47,5
	278	130,73	**300**	371	380	151	621	34	4,66	0,4	143	37	67	55,0

	142	66,78	**360**	517	368	152	694	30	5,05	0,3	153	37	57	44,0
	60	28,22	**420**	648	350	152	786	28	5,56	0,3	151	33	70	32,0
